# Supplementary material for: Dynalign II: common secondary structure prediction for RNA homologs with domain insertions
Source: Nucleic Acids Res. 2014 Nov 21;42(22):13939–48. doi: 10.1093/nar/gku1172 (PMC4267632; doi:10.1093/nar/gku1172)
Supplement: SUPPLEMENTARY DATA [file supp_gku1172_nar-02021-z-2014-File012.zip › manual/RNA_class/html/class_thermodynamics.html]

RNAstructure Classes: Thermodynamics Class Reference

- Main Page
- Classes
- Files

- Class List
- Class Hierarchy
- Class Members

# Thermodynamics Class Reference

Thermodynamics Class.
More...

`#include <thermodynamics.h>`

Inherited by RNA.

List of all members.

|  |  |
| --- | --- |
|  |  |
| Public Member Functions | |
|  | Thermodynamics (const bool ISRNA=true) |
| int | SetTemperature (double temperature) |
|  | Set the temperature of folding in K. |
| double | GetTemperature () |
|  | Get the current folding temperature in K. |
| int | ReadThermodynamic (const char \*pathname=NULL) |
|  | Function to read the thermodynamic parameters. |
| datatable \* | GetDatatable () |
| datatable \* | GetEnthalpyTable () |
| void | CopyThermodynamic (Thermodynamics \*thermo) |
|  | Copy thermodynamic parameters from an instance of Thermodynamics class. |
| bool | GetEnergyRead () |
|  | Return whether this instance of Thermodynamics has the paremters populated (either from disk or from another Thermodynamics class). |
|  | ~Thermodynamics () |
| Public Attributes | |
| bool | isrna |
| Protected Member Functions | |
| void | GetDat (char \*loop, char \*stackf, char \*tstackh, char \*tstacki, char \*tloop, char \*miscloop, char \*danglef, char \*int22, char \*int21, char \*coax, char \*tstackcoax, char \*coaxstack, char \*tstack, char \*tstackm, char \*triloop, char \*int11, char \*hexaloop, char \*tstacki23, char \*tstacki1n, char \*datapath, bool isRNA, bool isEnthalpy=false) |
| Protected Attributes | |
| bool | energyread |
|  | Access the thermodynamic parameter class an instance of datatable. |
| datatable \* | data |
| datatable \* | enthalpy |
| double | temp |

---

## Detailed Description

Thermodynamics Class.

The RNA class provides an encapsulation of the functions and struct for reading and storing thermodynamic parameters. This includes methods for changing folding temperatures This class is intended for use in inheritance for classes that provide functionality. 

---

## Constructor & Destructor Documentation

|  |  |  |  |  |  |
| --- | --- | --- | --- | --- | --- |
| Thermodynamics::Thermodynamics | ( | const bool | *ISRNA* = `true` | ) |  |

|  |  |  |  |  |
| --- | --- | --- | --- | --- |
| Thermodynamics::~Thermodynamics | ( |  | ) |  |

---

## Member Function Documentation

|  |  |  |  |  |  |
| --- | --- | --- | --- | --- | --- |
| void Thermodynamics::CopyThermodynamic | ( | Thermodynamics \* | *thermo* | ) |  |

Copy thermodynamic parameters from an instance of Thermodynamics class.

This is generally not needed because functions automatically populate the parameters from disk. It is helpful, however, when a large number of calculations with be performed because the parameters can then be read from disk only once. Note that the source Thermodynamics class must have been initialized with the "correct" ISRNA value and correct temperature. Return 0 if no error and non-zero errors can be parsed by GetErrorMessage() or GetErrorMessageString().

**Parameters:**
:   |  |  |  |
    | --- | --- | --- |
    |  | *thermo* | is a pointer to Thermodynamics class. That must have already called the ReadThermodynamics() function. |

|  |  |  |  |  |
| --- | --- | --- | --- | --- |
| void Thermodynamics::GetDat | ( | char \* | *loop*, |  |
|  |  | char \* | *stackf*, |  |
|  |  | char \* | *tstackh*, |  |
|  |  | char \* | *tstacki*, |  |
|  |  | char \* | *tloop*, |  |
|  |  | char \* | *miscloop*, |  |
|  |  | char \* | *danglef*, |  |
|  |  | char \* | *int22*, |  |
|  |  | char \* | *int21*, |  |
|  |  | char \* | *coax*, |  |
|  |  | char \* | *tstackcoax*, |  |
|  |  | char \* | *coaxstack*, |  |
|  |  | char \* | *tstack*, |  |
|  |  | char \* | *tstackm*, |  |
|  |  | char \* | *triloop*, |  |
|  |  | char \* | *int11*, |  |
|  |  | char \* | *hexaloop*, |  |
|  |  | char \* | *tstacki23*, |  |
|  |  | char \* | *tstacki1n*, |  |
|  |  | char \* | *datapath*, |  |
|  |  | bool | *isRNA*, |  |
|  |  | bool | *isEnthalpy* = `false` |  |
|  | ) |  |  | `[protected]` |

|  |  |  |  |  |
| --- | --- | --- | --- | --- |
| datatable \* Thermodynamics::GetDatatable | ( |  | ) |  |

This function is used during inheritance o provide access to the free energy change parameters. This function generates no error codes. (Error checking was done for this during construction).

**Returns:**
:   A pointer to datatable with free energy change parameters.

|  |  |  |  |  |
| --- | --- | --- | --- | --- |
| bool Thermodynamics::GetEnergyRead | ( |  | ) |  |

Return whether this instance of Thermodynamics has the paremters populated (either from disk or from another Thermodynamics class).

**Returns:**
:   A bool yjay indicates whether the parameters are populated (true = yes).

|  |  |  |  |  |
| --- | --- | --- | --- | --- |
| datatable \* Thermodynamics::GetEnthalpyTable | ( |  | ) |  |

This function is used to provide an enthalpy table. This function will return a NULL pointer if there is an error reading the tables from disk. It is important that programs check the status of the pointer before using it, i.e. make sure it is not NULL.

**Returns:**
:   A pointer to datatable with the enthalpy change parameters.

|  |  |  |  |  |
| --- | --- | --- | --- | --- |
| double Thermodynamics::GetTemperature | ( |  | ) |  |

Get the current folding temperature in K.

**Returns:**
:   A double that indicates the folding temperature in K.

|  |  |  |  |  |  |
| --- | --- | --- | --- | --- | --- |
| int Thermodynamics::ReadThermodynamic | ( | const char \* | *pathname* = `NULL` | ) |  |

Function to read the thermodynamic parameters.

This function depends on temp, the current temperature, to determine in the folding free energies need to be set to other than those read in files for 310.15 K. Return of zero => no error and a return of non-zero indicates error. Public functions that need the thermodynamic parameters call this function automatically. By default, the path to the thermodynamic paramaters is fetched from the $DATAPATH environment variable. If a specific path is needed, $DATAPATH is overridden by specifying the pathname explicitly here as a parameter.

**Returns:**
:   An int that indicates whether an error occured.

**Parameters:**
:   |  |  |  |
    | --- | --- | --- |
    |  | *pathname* | is a pointer to cstring that indicates the pathname to the thermodynamnic parameters. By default, this is NULL and the environment variable $DATAPATH is consulted to get this path. |

|  |  |  |  |  |  |
| --- | --- | --- | --- | --- | --- |
| int Thermodynamics::SetTemperature | ( | double | *temperature* | ) |  |

Set the temperature of folding in K.

This function allows the user to specify folding temperatures other than 310.15 K (37 degrees C). This changes folding free energy changes that would be returned for existing structures and would alter the set of structures predicted. When this function is called, the thermodynamic parameter files are immediately read from disk. These include both enthalpy parameters (.dh files) and free energy changes at 310.15 (.dat files). The files must either be at a location indicated by the $DATAPATH environment variable or in pwd. Changing the temperature only alters subsequent calculations. For example, if a structure prediction method has been called, the set of predicted structures are not changed at the time of a call to SetTemperature. Likewise, SetTemperature must be called before calling a structure prediction method if a temperature other than the 310.15 K default is desired. The function returns an error code where 0 is no error and non-zero errors can be parsed by by GetErrorMessage() or GetErrorMessageString() in an inheriting class.

---

## Member Data Documentation

datatable\* Thermodynamics::data `[protected]`

bool Thermodynamics::energyread `[protected]`

Access the thermodynamic parameter class an instance of datatable.

datatable\* Thermodynamics::enthalpy `[protected]`

bool Thermodynamics::isrna

double Thermodynamics::temp `[protected]`

---

The documentation for this class was generated from the following files:

- F:/RNAstructure-co-from-repository/RNAstructure/RNA\_class/thermodynamics.h- F:/RNAstructure-co-from-repository/RNAstructure/RNA\_class/thermodynamics.cpp

---

Generated on Tue Jul 30 10:52:29 2013 for RNAstructure Classes by 
 1.5.7.1
